# Do the Ticks of Birds at an Important Migratory Hotspot Reflect the Seasonal Dynamics of *Ixodes ricinus* at the Migration Initiation Site? A Case Study in the Danube Delta

**DOI:** 10.1371/journal.pone.0089378

**Published:** 2014-02-19

**Authors:** Attila D. Sándor, Daniel I. Mărcuţan, Gianluca D'Amico, Călin M. Gherman, Mirabela O. Dumitrache, Andrei D. Mihalca

**Affiliations:** Department of Parasitology and Parasitic Diseases, University of Agricultural Sciences and Veterinary Medicine Cluj-Napoca, Cluj-Napoca, Romania; University of Western Ontario, Canada

## Abstract

Migratory birds play important roles as distributors of ticks within and between continents. In the Old World, the most important migratory route of birds links Asia, Europe and Africa. During their migration, birds use various stopover sites, where they feed and rest and where ticks may attach or detach, creating new natural foci for vector-borne diseases. Danube Delta is one of the most important migration hotspots and so far no studies were focused on ticks of migratory birds herein. The aim of the present study was to assess the species diversity and seasonal dynamics of ticks parasitizing migratory birds in Danube Delta Biosphere Reserve. Migratory birds were trapped on Grindul Lupilor (44°41′N; 28°56′E) using mist nets during 4 migratory seasons (2 spring and 2 autumn) in 2011 and 2012. From each bird, all the ticks were collected and identified based on morphological features. Epidemiological parameters (prevalence, mean abundance, mean intensity) were calculated and all data were analysed statistically based on the season (spring and autumn), regional status of birds (migrants and breeding) and foraging behaviour (ground feeders, reed-bed feeders, foliage feeders). A total of 1434 birds (46 species) were captured. Ticks were found on 94 birds (10 species). Significantly more migratory birds hosted ticks, compared to resident birds. The 400 collected ticks belonged to four species: *Ixodes ricinus* (92.25%), *I. arboricola* (6.25%), *I. redikorzevi* (1.00%) and *Haemaphysalis punctata* (0.50%). A higher prevalence was found for *I. ricinus* in spring, with higher prevalence of nymphs in this season, while larvae occurred with the same prevalence in both seasons. Larval intensity was higher during spring and nymphs were more abundant during autumn. The seasonal differences in our study may be related not to the local seasonal dynamics of ticks, but on the seasonal dynamics at the site of migration initiation.

## Introduction

Infections caused by vector-borne pathogens are important emerging diseases all over the world [1], [2]. Most of them are zoonoses and represent one of the most important emerging health threats, both in the tropics, as well as at temperate latitudes [3], [4]. Their importance resides not only in incidence and virulence, but also in infecting a substantial part of the human population worldwide [5].

Arthropods feeding on both wildlife and humans are important links for pathogen transmission. The most important vectors and reservoir hosts of tick-borne pathogens and their mechanism of transmission are widely studied and mostly known [6]. However, the way these vectors (and their associated pathogens) are able to overcome geographical barriers, have recently become an important research topic. There are a number of examples for such emerging cases of pathogen transfer to previously unknown areas. One of the most dramatic models is the introduction of West Nile virus into the North American continent [7], or the arrival of novel viruses (i.e. Usutu virus, Paridae pox) to Europe [8], [9].. Although these cases are exceptional, even the smaller scale territorial leaps (i.e. inside a continent) are equally important, and the mechanisms beyond are largely unknown [4]. The rapidity of territorial transfer at such distances depends largely on the carrier vectors, vertebrates or invertebrates. Thus, animal species travelling large distances may speed-up the transfer. Birds are among the longest migrants and the number and biomass of migratory birds travelling between continents is the largest in the animal world [10]. Moreover, this migration happens twice every year, thus birds possess an extraordinary disseminating potential for attached ectoparasites (usually arthropods) or pathogens carried by them [11], [12].

Among ectoparasites of birds, ticks are responsible for hosting a significant number of human pathogens [13]. Ticks are the most important or exclusive vectors for pathogenic bacteria like *Borrelia burgdorferi* s.l. (causing Lyme disease), other *Borrelia* spp. (causing tick-borne relapsing fever), *Rickettsia* spp. (causing spotted fevers), *Ehrlichia* spp. (causing different types of ehrlichiosis), *Anaplasma* spp. (causing anaplasmosis), *Coxiella burnetii* (causing Q fever) or *Francisella tularensis* (causing tularaemia) [14], [15], [16]. Ticks may act also as vectors for a number of viral diseases like the tick-borne encephalitis, Crimean-Congo haemorrhagic fever or Colorado-tick fever [17], [18].

Wild birds are hosts for several species of ticks, contributing to the maintenance of their local populations in delimited geographic areas [19]. However, migratory birds play important roles as distributors of ticks within and between continents [20]. The most important migratory route of birds in the Old World (the Palearctic-African migratory pathway) links three continents: Asia, Europe and Africa [10]. Inside Europe, the migratory routes of individual species are diverse, with both North-South and West-East components. A common strategy of migrating birds is to use different stopover sites along their routes. At these sites, where birds feed and rest, ticks and other ectoparasites may attach and/or detach. New natural foci of tick borne disease may be created in this way [21]. The extensive wetland complex of the Danube Delta provides an internationally important stopover site for millions of birds, belonging to 300 different species, travelling annually to and from Northern Eurasia and Africa [10]. The location is also important for its diversity in tick species [22], [23] and for the presence of important human tick-borne pathogens like *Borrelia burgdorferi* s.l. [24], [25], [26], [27], *Anaplasma phagocytophilum* and *Coxiella burnetii* [28] or the Crimean-Congo haemorrhagic fever [29]. Moreover, Danube Delta has been considered an avian-influenza hotspot [30]. All these factors make Danube Delta a unique ecosystem for studying the role and importance of migratory birds in the ecology of ticks and their transport during migration.

The aim of this study was to assess the species diversity and seasonal dynamics of ticks parasitizing migratory birds in Danube Delta Biosphere Reserve, an important refuge for birds travelling between continents.

## Materials and Methods

Migratory birds were trapped in the Danube Delta, during 4 migratory seasons: 2 in spring (April) and 2 in autumn (October) in the years 2011 and 2012. Birds were captured using ornithological mist nets erected in different habitats around the ornithological laboratory at Grindul Lupilor, Tulcea, Romania (44°41′N; 28°56′E). The area is part of Danube Delta Biosphere Reserve; hence the field studies were carried out based on research permits, issued for each trapping season by the Research Authorization Department of the Danube delta Biosphere Reserve Administration. The studies were not performed on private land, or on other location requiring specific permissions. The research has been approved by the ethics committee of the University of Agricultural Sciences and Veterinary Medicine Cluj-Napoca. No other ethics approvals were required, as the field studies did not involve endangered or protected species and no animals were killed during the sample collection. Invasive methods were not used on the trapped birds.

The study area is a narrow land strip of NE-SW direction, between the brackish lakes of Goloviţa-Zmeica and Sinoie, in the SW part of the Danube Delta. It is covered mostly by reed beds (*Phragmites australis*) and reed-mace stands (*Typha* spp.), with small patches of marshy vegetation (mostly *Carex* spp, *Juncus* spp.) and dry grasslands on sand. The bird trapping site was located within a 3.8 km long strip of woody vegetation of variable width (mostly under 20 m, maximum 112 m) made by *Salix* spp., *Elaeagnus angustifolia*, *Hippophae rhamnoides* and a few white poplars (*Populus alba*). The fauna of the area is rich in breeding water birds and passerines breeding in reed-beds, with high numbers of migrant passerines using the area as short-time stop-over location in both migratory seasons [31]. The trapping lasted for one week in each season and occurred in April and October in both years, targeting the migration peak of small to medium sized passerines in the region. We used 12 mist nets (5 shelf type, 12 m long each, Ecotone Inc.), erected in reed bed, reed-mace stands and between woody vegetation. Nets were arranged in a way to target most passerines landing on the ground and were controlled on hourly basis during daylight and one hour after sunset. All birds were extracted and identified to species (and whenever possible aged and sexed) and ringed with individually numbered metal rings. Prior to their release, birds were carefully inspected by the same observer (ADS) for the presence of ticks. Ticks were collected from the body of birds with a fine forceps and preserved in absolute ethanol for later examination using a separate vial for each bird. All ticks were collected. Ticks were identified under a stereo microscope to species, developmental stage and sex in adults, based on morphological features, using dichotomous keys [32], [33].

For studying the seasonal distribution of ticks, the two trapping seasons (spring and autumn) were considered separately. Bird species were grouped according to their status in the region: migrants (occurring for short periods lasting from a few days to a few weeks in spring and/or autumn) and breeding birds (either migratory or resident, spending the boreal summer in the region). To evaluate the differences between tick-attachment rates, birds were also grouped according to their foraging behaviour: (1) ground feeders (species feeding only or mostly on the soil layer), (2) reed-bed feeders (species using primarily the reed and reed-mace stands) and (3) foliage feeders (species using the bushes and canopy of trees). The European Barn Swallow, *Hirundo rustica*, the only aerial feeder caught was omitted from the statistical analyses.

Three epidemiological parameters were calculated for each bird species, each tick developmental stage and total number of ticks: prevalence (% of birds with ticks), mean abundance (mean number of ticks per total examined birds) and mean intensity (mean number of ticks per total infested birds). The difference of prevalence was statistically analysed by chi-squared (*χ^2^*) independence, while for testing the effect of season, feeding regime and status we used Generalized Linear Model (GLZ). A *p* value of <0.05 was statistically significant. All statistical analyses were performed using the **Epi Info 2000** (version 3.5.1) and **Statsoft Statistica** version 7.0 (**Statsoft** Inc., Tulsa, Oklahoma, USA) softwares.

## Results

A total of 1435 birds in 49 species of Passeriformes and 3 non-Passeriformes bird species (Table 1) were captured. Most birds were caught during autumn (n = 1168; 81.39%), with slightly more migrants caught in this season (54% vs. 67%). No differences were noticed between the percentages of different feeding groups. Recapture of ringed birds was rare, accounting for 17 birds (1.18%) of 4 species, all being instraseasonal, local retraps. No local retraps had ticks, so they were excluded from further statistical analyses.

**Table 1:** Ticks present on birds investigated in the Danube Delta.

Host species	Number of birds captured	No tick infested/%	*I. ricinus*			*I. arboricola*		*I. redikorzevi*	*H. punctata*
			Larvae	Nymphs	Females	Larvae	Nymphs	Nymphs	Nymphs
*Accipiter nisus*	7	0/0							
*Acrocephalus agricola*	6	0/0							
*Acrocephalus arundinaceus*	3	0/0							
*Acrocephalus melanopogon*	2	0/0							
*Acrocephalus palustris*	5	0/0							
*Acrocephalus schoenobaenus*	10	0/0							
*Acrocephalus scirpaceus*	52	0/0							
*Anthus trivialis*	3	0/0							
*Asio otus*	1	0/0							
*Carduelis carduelis*	1	0/0							
*Carduelis chloris*	1	0/0							
*Carduelis spinus*	9	0/0							
*Coccothraustes coccothraustes*	1	0/0							
*Certhia familiaris*	1	0/0							
*Coturnix coturnix*	1	0/0							
*Cyanistes caeruleus*	30	2/6.9	1				1		
*Emberiza schoeniclus*	11	1/9.1	1						
*Erithacus rubecula*	352	28/7.8*	97	65	1	5	3	2	
*Ficedula albicollis*	5	0/0							
*Ficedula hypoleuca*	10	1/10.0	2						
*Ficedula parva*	17	0/0							
*Fringilla coelebs*	25	0/0							
*Hirundo rustica*	8	0/0							
*Lanius collurio*	1	0/0							
*Locustella luscinioides*	3	0/0							
*Luscinia megarhynchos*	1	0/0							
*Motacilla alba*	9	0/0							
*Muscicapa striata*	2	0/0							
*Panurus biarmicus*	306	2/0.6*		1				1	
*Parus major*	16	8/50.0*	4	7			3	1	
*Passer domesticus*	17	0/0							
*Phoenicurus ochruros*	18	0/0							
*Phoenicurus phoenicurus*	7	0/0							
*Phylloscopus collybita*	96	1/1.0		1					
*Phylloscopus trochilus*	8	0/0							
*Pica pica*	3	0/0							
*Prunella modularis*	7	0/0							
*Regulus regulus*	47	2/4.2	1	1					
*Remiz pendulinus*	5	1/20.0					1		
*Saxicola rubetra*	2	0/0							
*Saxicola torquata*	2	0/0							
*Sturnus vulgaris*	15	0/0							
*Sylvia atricapilla*	13	0/0							
*Sylvia borin*	1	0/0							
*Sylvia curruca*	31	0/0							
*Troglodytes troglodytes*	29	0/0							
*Turdus merula*	142	43/30.3	35	95	7		6		2
*Turdus philomelos*	91	6/6.6	40	10		5	1		
*Upupa epops*	2	0/0							
**Total**	**1435**	**95**	**181**	**180**	**8**	**10**	**15**	**4**	**2**

* Co-infection with *I. ricinus* and *I. redikorzevi*.

Ticks were found on 95 birds belonging to 11 species. A total of 400 ticks were collected and identified as larvae (n = 191; 47.75%), nymphs (n = 201; 50.25%) or adult females (n = 8; 2%). No adult males were recorded. The ticks belonged to four species (*Ixodes ricinus*, *I. arboricola*, *I. redikorzevi* and *Haemaphysalis punctata*). All ticks were collected from the head, with most found around the bill, ears, nape and crown. *Ixodes ricinus* was the most common tick (369 ticks, 92.25% of the total collected), with a total of 181 larvae, 180 nymphs and 8 females, parasitizing 79 birds of 10 species (Table 2). *Ixodes arboricola* (25 ticks, 6.25% of the total collected) was found on 14 birds of 6 species, with 10 larvae and 15 nymphs collected. *Ixodes redikorzevi* (4 ticks, 1.00% of the total collected), was found in 4 birds (3 species), with 4 nymphs, while *H. punctata* was found parasitizing one host, with 2 nymphs (2 ticks, 0.50% of the total collected). Polyspecific parasitism was rare, with only three instances of *I. ricinus* and *I. redikorzevi* occurring on the same bird (each of a different species).

**Table 2:** Prevalence and mean intensity (MI) of Ixodes ricinus on birds captured in Danube Delta.

	Eurasian Robin, *Erithacus rubecula*	Common Blackbird, *Turdus merula*	Song Trush, *Turdus philomelos*	Common Reed Bunting, *Emberiza schoeniclus*	Bearded Reedling, *Panurus biarmicus*	Blue Tit, *Cyanistes caeruleus*	European Pied Flycatcher, *Ficedula hypoleuca*	Great Tit, *Parus major*	Common Chiffchaff, *Phylloscopus collybita*	Goldcrest, *Regulus regulus*
**Foraging habitat**	**Ground**			**Reed**		**Foliage**				
**Status**	M	M	M	R	R	R	M	R	M	M
**Birds examined in spring**	40	22	3	4	0	3	10	1	4	0
**Birds infested in spring**	4	18	1	1	0	0	1	0	0	0
**Birds examined in autumn**	312	120	88	7	306	27	0	15	92	47
**Birds infested in autumn**	19	21	4	0	1	1	0	5	1	2
**Total examined birds**	352	140	91	11	306	29	10	16	96	47
**Total birds infested (prevalence)**	23 (6.5)	39 (27.8)	5 (5.5)	1 (9.1)	1 (0.3)	1 (3.4)	1 (10.0)	5 (31.2)	1 (1.0)	2 (4.2)
**No. of larvae spring (MI)**	0 (0)	6(1.2)	0	1(1)	0	0	2(2)	0	0	0
**No. of larvae autumn (MI)**	97 (8.08)	29(4.1)	40(13.3)	0	0	1(1)	0	4(4)	0	0
**No. of larvae total (MI)**	97(5.1)	35(2.9)	40(13.3)	1(1)	0	1	2	4(4)	0	0
**No. of nymphs spring (MI)**	5 (1.25)	75(4.41)	2(2)	0	0	0	0	0	0	0
**No. of nymphs autumn (MI)**	60(7.5)	20(1.66)	8(4)	0	1(1)	0	0	7(1.75)	1(1)	2(1)
**No. of nymphs total (MI)**	66(2.8)	94(5.5)	10(3.3)	0	1	0	0	7(1.75)	1(1)	2(1)
**No. of females spring (MI)**	0	3(1)	0	0	0	0	0	0	0	0
**No. of females autumn (MI)**	1 (1)	4(1)	0	0	0	0	0	0	0	0
**No. of females total (MI)**	1(1)	7(1)	0	0	0	0	0	0	0	0
**No. of ticks spring (MI)**	5(1.25)	82 (4.55)	2(2)	1(1)	0	0	2(2)	0	0	0
**No. of ticks autumn (MI)**		53(2.5)	48(12)	0	1(1)	1(1)	0	11(2.2)	1(1)	2(1)
**No. of ticks total (MI)**	164(7.1)	136(3.5)	50(10)	1(1)	1(1)	1(1)	2(2)	11(2.2)	1(1)	2(1)
**Prevalence larvae**	0.05	0.08	0.03	0.09	0	0.03	0.2	0.06	0	0
**Prevalence nymphs**	0.06	0.2	0.03	0	0.003	0.000	0	0.25	0.1	0.4
**Prevalence females**	0.002	0.05	0	0	0	0	0	0	0	0

M - Migratory; R - Resident.

There were no differences between: (1) the prevalence and intensity of tick infestation between corresponding seasons in the two years of the study; (2) between the age or sex groups within of the same host species (Table 2.).

There were differences among tick distribution patterns, with *I. ricinus* and *I. arboricola* occurring both in spring and autumn, while *I. redikorzevi* and *H. punctata* were found only in autumn. As the prevalence of *I. arboricola*, *I. redikorzevi* and *H. punctata* were low and their occurrences were accidental, they were omitted from further statistical analyses.

A higher prevalence was found for *I. ricinus* in spring (0.35 vs. 0.09), with higher prevalence of nymphs (0.35 vs. 0.04) in this season, while larvae occurred with the same prevalence in both seasons. Only one species had significant differences, *Turdus merula*, with significantly higher overall prevalence for spring (GLZ, with binomial distribution of the dependent variable, *χ^2^* = 34.79, df = 1, P<0.0001), explained by the signifcantly higher nymphal prevalence in this season (Mann-Whitney U Test, Z = −5.29, n1 = 120, n2 = 22, P<0.0001).

The mean intensity of parasitism, regardless the developmental stage, was not different among seasons. Significantly more migratory birds hosted ticks, compared to resident birds (*χ^2^* = 22.70, df = 1, P<0.01), with higher intensity of all stages in migratory birds (3.6 in migratory birds vs. 1.75 in resident species).

Birds feeding on the ground were found to hold the highest prevalence of ticks, however this difference was significant only raported to birds feeding in reed (*χ^2^* = 12.18848 df = 1, P<0.001), while the lowest prevalence was detected among birds feeding in the reed. There were no differences among the distribution patterns of different development stages among the different feeding groups. However, higher intensities of larvae and nymphs were found in the case of ground feeders regardless the season (see Tab 2.).

## Discussion and Conclusions

Four different tick species were found parasitizing migratory birds in the Danube Delta, with *I. ricinus* evidently dominating the community parasitic on birds captured during the spring (northward) and autumn (southward) migration. *Ixodes arboricola* occurred with a much lower prevalence and intensity in both seasons, while *I. redikorzevi* and *H. punctata* were found only during the autumn. These results are similar with studies from other areas important for migratory birds with *I. ricinus* being considered the most common tick species of Palaearctic-African migratory birds [11], [19], [34], [35], [36]. This species is widespread and common in Romania [37], occurring commonly on birds as well [22] and it is abundant in the region [37].

The presence of *I. arboricola* is typical for birds, with most occurrences found on ground feeding species, especially in southern [19] and central part of Europe [33]. *Haemaphysalis punctata* is widely reported from birds, usually with a higher prevalence, while *I. redikorzevi* is a tick with an eastern distribution in Europe (occurring mainly in Asia), feeding primarily on small mammals [38]. Reports of this later species are rare on birds and most cases relate to migrants from Asia or Eastern Europe to Africa [39], [40].

Tick abundance was not similar in the two migratory seasons. A higher prevalence and intensity was associated to the northward migrating birds, in line with previous research [11], [20], [21], [41]. Stage structure of populations of parasitic ticks collected from wild birds was significantly in favour of nymphs in spring. *Ixodes ricinus* is known to have different seasonal population dynamics, depending on local environmental conditions through its relatively vast distribution range [42]. The seasonal differences in our study are probably related not as much to the local seasonal dynamics of ticks, but rather on the seasonal dynamics at the site of migration initiation. A recent study on migratory birds [36] found that in Southern Europe, during early spring, the nymphs are more abundant than larvae. Moreover, a study of population seasonal dynamics of questing *I. ricinus* in the same region, found that nymph are more abundant in winter and early spring and larvae predominate during autumn [43]. This is in accordance with our results, as we found the greatest abundance of nymphs during spring on birds which regularly overwinter in the Mediterranean region [44].

On the other hand, in birds captured during the autumn, when the initiation of migration was in North, North-Eastern Europe or North-Western Asia [20], the abundance of ticks was significantly lower in each developmental stage and host species. In those areas, this period of the year is characterized by a lower density of questing ticks [42].

Ground feeding passerines seem to be the most important hosts for the majority of tick species and developmental stages [12], [19], [35]. In the case of Danube Delta, ground feeders had a fivefold higher prevalence and tenfold higher intensity compared to canopy feeders or reed-dwellers. Moreover, ground feeding species studied in the Danube Delta (e.g. Blackbird, *Turdus merula* and European Robin, *Erithacus rubecula*) are among the most urbanised species also, with high population densities inside human settlement [45], thus posing the highest risk of tick transfer between areas important for human population.

Generally, migratory bird species carried more ticks than residents, with urbanised birds being the most parasitized. This phenomenon might be important for the transfer of ticks (and associated pathogens) among distant locations [46], but more complex studies are required to demonstrate this.

While resident birds are important in maintaining tick populations regionally [47], migrants bring in each migratory season high numbers of ticks from the breeding (presumably NE Europe and Asia) or wintering areas (Africa) to Europe [20], [39], [40] or may transfer ticks among different stop-over sites.

## References

[1] Randolph SE (2010) To what extent has climate change contributed to the recent epidemiology of tick-borne diseases? Vet Parasitol 167: 92–94. 19833440 10.1016/j.vetpar.2009.09.011

[2] Smith KF, Guégan JF (2010) Changing geographic distributions of human pathogens. Annu Rev Ecol Evol S 41: 231–250.

[3] Havelaar AH, van Rosse F, Bucura C, Toetenel MA, Haagsma JA, et al (2010) Prioritizing emerging zoonoses in the Netherlands. PLoS One 5: e13965. 21085625 10.1371/journal.pone.0013965 PMC2981521

[4] Lindgren E, Andersson Y, Suk JE, Sudre B, Semenza JC (2012) Monitoring EU emerging infectious disease risk due to climate change. Science 336: 418–419. 22539705 10.1126/science.1215735

[5] Woolhouse M, Gaunt E (2007) Ecological origins of novel pathogens. Crit Rev Microbiol 33: 231–342. 18033594 10.1080/10408410701647560

[6] Franke J, Hildebrandt A, Dorn W (2013) Exploring gaps in our knowledge on Lyme borreliosis spirochaetes - Updates on complex heterogeneity, ecology, and pathogenicity. Ticks Tick Borne Dis 4: 11–25. 23246041 10.1016/j.ttbdis.2012.06.007

[7] Añez G, Grinev A, Chancey C, Ball C, Akolkar N, et al (2013) Evolutionary dynamics of West Nile virus in the United States, 1999–2011: phylogeny, selection pressure and evolutionary time-scale analysis. PLoS Negl Trop Dis 7: e2245. 23738027 10.1371/journal.pntd.0002245 PMC3667762

[8] Becker N, Jöst H, Ziegler U, Eiden M, Höper D, et al (2012) Epizootic emergence of Usutu virus in wild and captive birds in Germany. PLoS One 7: e32604. 22389712 10.1371/journal.pone.0032604 PMC3289667

[9] Lachish S, Lawson B, Cunningham AA, Sheldon BC (2012) Epidemiology of the emergent disease Paridae pox in an intensively studied wild bird population. PLoS One 7: e38316. 23185230 10.1371/journal.pone.0038316 PMC3504069

[10] Newton I (2008) The migration ecology of birds. London: Academic Press. 984 p.

[11] Hildebrandt A, Franke J, Meier F, Sachse S, Dorn W, et al (2010) The potential role of migratory birds in transmission cycles of *Babesia* spp., *Anaplasma phagocytophilum*, and *Rickettsia* spp. Ticks Tick Borne Dis 1: 105–107. 21771516 10.1016/j.ttbdis.2009.12.003

[12] Hasle G, Bjune GA, Midthjell L, Røed KH, Leinaas HP (2011) Transport of *Ixodes ricinus* infected with *Borrelia* species to Norway by northward-migrating passerine birds. Ticks Tick Borne Dis 2: 37–43. 21771535 10.1016/j.ttbdis.2010.10.004

[13] de la Fuente J, Estrada-Peña A (2012) Ticks and tick-borne pathogens on the rise. Ticks Tick Borne Dis 3: 115–116. 10.1016/j.ttbdis.2012.03.001 22609243

[14] Elfving K, Olsen B, Bergström S, Waldenström J, Lundkvist A, et al (2010) Dissemination of Spotted Fever Rickettsia agents in Europe by migrating birds. PLoS One 5: e8572. 20052286 10.1371/journal.pone.0008572 PMC2797135

[15] Edouard S, Koebel C, Goehringer F, Socolovschi C, Jaulhac B, et al (2012) Emergence of human granulocytic anaplasmosis in France. Ticks Tick Borne Dis 3: 403–405. 23182272 10.1016/j.ttbdis.2012.10.002

[16] Oteo JA, Portillo A (2012) Tick-borne rickettsioses in Europe. Ticks Tick Borne Dis 3: 271–278. 23177355 10.1016/j.ttbdis.2012.10.035

[17] Süss J (2011) Tick-borne encephalitis 2010: epidemiology, risk areas, and virus strains in Europe and Asia - an overview. Ticks Tick Borne Dis 2: 2–15. 21771531 10.1016/j.ttbdis.2010.10.007

[18] Kunze U (2012) ISW-TBE (2012) Tick-borne encephalitis (TBE): an underestimated risk…still: Report of the 14th Annual Meeting of the International Scientific Working Group on Tick-Borne Encephalitis (ISW-TBE). Ticks Tick Borne Dis 3: 197–201. 22765977 10.1016/j.ttbdis.2012.03.007

[19] Norte AC, de Carvalho IL, Ramos JA, Gonçalves M, Gern L, et al (2012) Diversity and seasonal patterns of ticks parasitizing wild birds in western Portugal. Exp Appl Acarol 58: 327–339. 22669280 10.1007/s10493-012-9583-4

[20] Hoogstraal H, Kaiser MN, Traylor MA, Gaber S, Guindy E (1961) Ticks (Ixodoidea) on birds migrating from Africa to Europe and Asia. Bull World Health Organ 24: 197–212. 13715709 PMC2555510

[21] Olsén B, Jaenson TGT, Bergström SA (1995) Prevalence of *Borrelia burgdorferi* sensu lato-infected ticks on migrating birds. Appl Environ Microbiol 61: 3082–3087. 7487041 10.1128/aem.61.8.3082-3087.1995 PMC167585

[22] Mihalca AD, Dumitrache MO, Magdaş C, Gherman CM, Domşa C, et al (2012) Synopsis of the hard ticks (Acari: Ixodidae) of Romania with update on host associations and geographical distribution. Exp Appl Acarol 58: 183–206. 22544174 10.1007/s10493-012-9566-5

[23] Mihalca AD, Dumitrache MO, Sándor AD, Magdaş C, Oltean M, et al (2012) Tick parasites of rodents in Romania: host preferences, community structure and geographical distribution. Parasit Vectors 5: 266. 23171665 10.1186/1756-3305-5-266 PMC3514150

[24] Majláthová V, Majláth I, Hromada M, Tryjanowski P, Bona M, et al (2008) The role of the sand lizard (*Lacerta agilis*) in the transmission cycle of *Borrelia burgdorferi* sensu lato. Int J Med Microbiol 298: 161–167.17702653

[25] Kiss T, Cadar D, Krupaci AF, Bordeanu A, Brudaşcă GF, et al (2011) Serological reactivity to *Borrelia burgdorferi* sensu lato in dogs and horses from distinct areas in Romania. Vector Borne Zoonotic Dis 11: 1259–1262. 21612524 10.1089/vbz.2010.0254

[26] Gherman C, Sándor AD, Kalmár Z, Marinov M, Mihalca AD (2012) First report of *Borrelia burgdorferi* sensu lato in two threatened carnivores: the Marbled polecat, *Vormela peregusna* and European mink, *Mustela lutreola* (Mammalia: Mustelidae). BMC Vet Res 8: 137. 22901862 10.1186/1746-6148-8-137 PMC3514366

[27] Kalmár Z, Mihalca AD, Dumitrache MO, Gherman CM, Magdaş C, et al (2013) Geographical distribution and prevalence of *Borrelia burgdorferi* genospecies in questing *Ixodes ricinus* from Romania: a countrywide study. Ticks Tick Borne Dis in press. 10.1016/j.ttbdis.2013.04.007 23890805

[28] Paştiu AI, Matei IA, Mihalca AD, D'Amico G, Dumitrache MO, et al (2012) Zoonotic pathogens associated with *Hyalomma aegyptium* in endangered tortoises: evidence for host-switching behaviour in ticks? Parasit Vectors 5: 301. 23273169 10.1186/1756-3305-5-301 PMC3564739

[29] Ceianu CS, Panculescu-Gatej RI, Coudrier D, Bouloy M (2012) First serologic evidence for the circulation of Crimean-Congo Hemorrhagic Fever Virus in Romania. Vector Borne Zoonotic Dis 12: 718–721. 22897346 10.1089/vbz.2011.0768

[30] Gaidet N, Ould El Mamy AB, Cappelle J, Caron A, Cumming GS, et al (2012) Investigating avian influenza infection hotspots in old-world shorebirds. PLoS One 7: e46049. 23029383 10.1371/journal.pone.0046049 PMC3460932

[31] Iojă CI, Pătroescu M, Rozylowicz L, Popescu VD, Vergheleþ M, et al (2010) The efficacy of Romania's protected areas network in conserving biodiversity. Biol Cons 143: 2468–2476.

[32] Feider Z (1965) Arachnida. Acaromorpha, Suprafamily Ixodoidea (Ticks), Fauna of the Peoples Republic of Romania [in Romanian]. Bucharest: Editura Academiei Republicii Populare Române. 404 p.

[33] Nosek J, Sixl W (1972) Central-European ticks (Ixodoidea). Mitt Abt Zool Landesmus Joanneum 1: 61–92.

[34] Movila A, Gatewood A, Toderas I, Duca M, Papero M, et al (2008) Prevalence of *Borrelia burgdorferi* sensu lato in *Ixodes ricinus* and *I. lividus* ticks collected from wild birds in the Republic of Moldova. Int J Med Microbiol 298: 149–153.

[35] Palomar AM, Santibáñez P, Mazuelas D, Roncero L, Santibáñez S, et al (2012) Role of birds in dispersal of etiologic agents of tick-borne zoonoses, Spain, 2009. Emerg Infect Dis 18: 1188. 22709801 10.3201/eid1807.111777 PMC3376802

[36] Falchi A, Dantas-Torres F, Lorusso V, Malia E, Lia RP, et al (2012) Autochthonous and migratory birds as a dispersion source for *Ixodes ricinus* in southern Italy. Exp Appl Acarol 58: 167–174. 22610454 10.1007/s10493-012-9571-8

[37] Mihalca AD, Gherman CM, Magdaş C, Dumitrache MO, Györke A, et al (2012) *Ixodes ricinus* is the dominant questing tick in forest habitats in Romania: the results from a countrywide dragging campaign. Exp Appl Acarol 58: 175–182. 22547023 10.1007/s10493-012-9568-3

[38] Kolonin GV (2009) Fauna of Ixodid ticks of the world (Acari, Ixodidae). Available: http://www.kolonin.org. Accessed 10 May 2013.

[39] Hoogstraal H, Traylor MA, Gaber S, Malakatis G, Guindy E, et al (1964) Ticks (Ixodidae) on migrating birds in Egypt, spring and fall 1962. Bull World Health Organ 30: 355–367. 14163959 PMC2554818

[40] Kaiser MN, Hoogstraal H, Watson GE (1974) Ticks (Ixodoidea) on migrating birds in Cyprus, fall 1967 and spring 1968, and epidemiological considerations. Bull Entomol Res 64: 97–110.

[41] Poupon MA, Lommano E, Humair PF, Douet V, Rais O, et al (2006) Prevalence of *Borrelia burgdorferi* sensu lato in ticks collected from migratory birds in Switzerland. Appl Environ Microbiol 72: 976–979. 16391149 10.1128/AEM.72.1.976-979.2006 PMC1352204

[42] Korenberg EI (2000) Seasonal population dynamics of *Ixodes* ticks and tick-borne encephalitis virus. Exp Appl Acarol 24: 665–681. 11227825 10.1023/a:1010798518261

[43] Dantas-Torres F, Otranto D (2013) Seasonal dynamics of *Ixodes ricinus* on ground level and higher vegetation in a preserved wooded area in southern Europe. Vet Parasitol 192: 253–258. 23092681 10.1016/j.vetpar.2012.09.034

[44] Tellería JL, Ramírez Á, Galarza A, Carbonell R, Pérez-Tris J, et al (2009) Do migratory pathways affect the regional abundance of wintering birds? A test in northern Spain. J Biogeogr 36: 220–229.

[45] Møller AP, Diaz M, Flensted-Jensen E, Grim T, Ibáñez-Álamo JD, et al (2012) High urban population density of birds reflects their timing of urbanization. Oecologia 170: 867–875. 22588633 10.1007/s00442-012-2355-3

[46] Altizer S, Bartel R, Han BA (2011) Animal migration and infectious disease risk. Science 331: 296–302. 21252339 10.1126/science.1194694

[47] Marsot M, Henry PY, Vourc'h G, Gasqui P, Ferquel E, et al (2012) Which forest bird species are the main hosts of the tick, *Ixodes ricinus*, the vector of *Borrelia burgdorferi* sensu lato, during the breeding season? Int J Parasitol 42: 781–788. 22732161 10.1016/j.ijpara.2012.05.010

